# Myofascial edema of gastrocnemius: A prominent MRI characteristic in dermatomyositis patients with anti‐transcriptional intermediate factor 1‐γ antibody

**DOI:** 10.1111/cns.14647

**Published:** 2024-02-22

**Authors:** Chao Zhen, Bing Zhao, Jingzhen He, Li Wei, Chuanzhu Yan, Tingjun Dai, Ying Hou

**Affiliations:** ^1^ Research Institute of Neuromuscular and Neurodegenerative Diseases and Department of Neurology Qilu Hospital of Shandong University Jinan China; ^2^ Department of Neurology, Qingdao Hospital University of Health and Rehabilitation Sciences (Qingdao Municipal Hospital) Qingdao China; ^3^ Department of Radiology Qilu Hospital of Shandong University Jinan Shandong China; ^4^ Department of Central Laboratory and Mitochondrial Medicine Laboratory, Qilu Hospital (Qingdao), Cheeloo College of Medicine Shandong University Qingdao China; ^5^ Brain Science Research Institute, Shandong University Jinan China

**Keywords:** anti‐TIF1‐γ dermatomyositis, fatty replacement, magnetic resonance imaging, muscle edema, myofascial edema

## Abstract

**Aims:**

This study was designed to evaluate the magnetic resonance imaging (MRI) patterns of the lower limb muscles in dermatomyositis (DM) with anti‐transcriptional intermediate factor 1‐γ (anti‐TIF1‐γ) antibody.

**Methods:**

This retrospective, observational, cross‐sectional study enrolled 12 adult DM patients with anti‐TIF1‐γ antibody. Muscles were assessed for fascial edema, subcutaneous‐tissue edema, muscle edema, and fatty replacement. These features were analyzed in relation to clinical characteristics.

**Results:**

All 12 patients underwent hip and thigh MRI, and 8 completed calf MRI. All patients showed myofascial edema, muscle edema, and fatty replacement, and 8 out of 12 further exhibited subcutaneous‐tissue edema. Specifically, myofascial edema of the gastrocnemius was observed in all patients (8/8). The vastus intermedialis and vastus lateralis muscles showed the most severe muscle edema, whereas the caput breve of the biceps femoris, semitendinosus, and soleus muscles exhibited the most severe fatty replacement. Although only 1 patient exhibited asymmetric muscle weakness, 9 showed asymmetric muscle edema, and 10 showed asymmetric fatty replacement. Changes in muscle edema positively correlated with creatine kinase (CK) levels.

**Conclusions:**

Myofascial edema of gastrocnemius was a prominent characteristic of anti‐TIF1‐γ‐positive DM. Early detection of muscle edema, as well as CK levels, may be helpful for monitoring disease activity.

## Graphical abstract

All patients with anti‐TIF‐1γ antibody showed myofascial edema, muscle edema, and fatty replacement of lower limb muscles. Myofascial edema of gastrocnemius was a prominent characteristic of this population, and muscle edema could serve as a sensitive biomarker for monitoring disease activity.

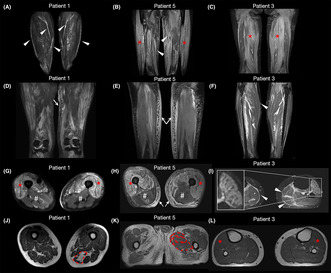

## 1 INTRODUCTION

Dermatomyositis (DM) is a specific subgroup of idiopathic inflammatory myopathy (IIM) characterized by proximal muscle weakness and skin lesions.^1^, ^2^ Anti‐transcriptional intermediary factor 1‐γ (TIF1‐γ) antibody is considered a DM‐specific antibody with a unique clinico‐pathological phenotype.^3^, ^4^ Specifically, adult anti‐TIF1‐γ antibody‐positive patients can be clinically characterized by proximal muscle weakness, erythematous skin rashes, malignancy, and dysphagia. Further, they often exhibit perifascicular atrophy, punched‐out fiber, and major histocompatibility complex (MHC) class expression with perifascicular enhancement on muscle biopsy.^5^, ^6^, ^7^, ^8^

Muscle magnetic resonance imaging (MRI), the gold standard technique for imaging studies of muscle diseases, has previously been used to detect unique patterns of fascial edema, muscle edema, and fatty replacement in different types of IIM.^9^, ^10^, ^11^, ^12^ Moreover, it can be used to evaluate disease activity and severity, monitoring therapy response, and identifying target site for biopsy.^13^, ^14^, ^15^, ^16^ Nevertheless, the pattern of muscle involvement by MRI and its association with disease activity in DM with anti‐TIF1‐γ antibody is still warranted. In this study, we analyzed the MRI changes of the lower limbs in a Chinese case series of anti‐TIF1‐γ antibody‐positive patients. We further investigated the association between the MRI features and clinical characteristics.

## 2 MATERIALS AND METHODS

### 2.1 Study design and patients

This was a retrospective, observational, and cross‐sectional study which enrolled 12 adult DM patients with anti‐TIF1‐γ antibody. Patients were identified from the Department of Neurology at Qilu Hospital between April 2017 and February 2022. All patients were diagnosed with DM in accordance with the 239th European Neuromuscular Centre International Workshop on DM.^4^ This study was approved by the Ethics Committee of Qilu Hospital (Qingdao). All patients or their guardians provided written informed consent.

### 2.2 Clinical and laboratory data

Relevant clinical data, including demographics, clinical course, neurological examinations, laboratory data, histopathological data, and treatment regimens, were collected. All patients underwent full screening for malignancy during the diagnostic workup period. Muscle strength was evaluated using the ordinal six‐point (0–5) manual muscle testing (MMT) scale, and asymmetric muscle weakness was defined as no less than one grade measured by MMT between the two sides of the same muscle group.^17^ Patients were followed up about every 2 months after diagnosis. The follow‐up time for each patient is added to Table 1. They were primarily followed up for symptoms and signs of muscle weakness, recovery of daily living ability, creatine kinase (CK) levels, and disease recurrence. The MMT grade of the weakest muscle group was used to determine the severity of weakness. Treatment outcomes were graded as follows: no improvement, mild improvement (1 grade in 1–2 muscle groups, continuously requiring help with walking and daily life activities), moderate improvement (>1 grade throughout several muscular groups, requiring little help walking and performing daily activities), marked improvement (symptoms and signs of mild muscle weakness but no functional restriction), or return to baseline (no symptoms or signs of muscle weakness).^18^ According to the schedule, patients who had disease recurrence required reexamination of muscle MRI again and adjustments for medical intervention. The research flowchart of our study is shown in Figure S1.

**TABLE 1:** Clinico‐pathological features of 12 anti‐TIF1‐γ‐positive adult DM patients.

Patient	Pt 1	Pt 2	Pt 3	Pt 4	Pt 5	Pt 6	Pt 7	Pt 8	Pt 9	Pt 10	Pt 11	Pt 12
Sex	F	F	F	F	F	M	F	F	F	F	F	F
Age (years)	71	77	53	66	76	65	47	51	19	73	49	48
Age of onset (years)	71	76	52	65	76	65	47	49	19	72	48	47
Onset to the final diagnosis (months)	2	12	12	18	6	3	5	24	1.5	0.7	12	18
CK (U/L)	3905	782	211	32	530	3366	146	892	824	544	36	315
Muscle manifestations												
MMT of the weakest muscle group	4	2	4	0	2	3	4	3	4	3	4	4
Symmetry	Y	Y	Y	Y	N	Y	Y	Y	Y	Y	Y	Y
Dysphagia	N	Y	Y	N	Y	Y	N	Y	Y	Y	N	Y
Malignancy	Ovarian cancer	Lymphoma of ileocecal region	Endometrial carcinoma	N	N	N	N	N	N	N	N	N
Obvious inflammatory cell infiltration	N	Y	Y	Y	N	Y	Y	Y	Y	Y	N	N
Perifascicular necrosis	N	N	N	N	N	Y	N	N	N	N	N	N
Perifascicular atrophy	N	N	Y	Y	N	N	Y	Y	N	N	Y	N
Autoimmune markers expression with perifascicular enhancement												
MHC‐I	N	N	Y	N	N	N	Y	Y	N	N	Y	N
MHC‐II	N	N	Y	N	Y	N	N	N	N	Y	Y	N
MAC‐Sac	N	N	Y	N	N	Y	N	N	N	N	N	N
MAC‐Cap	N	N	N	N	Y	N	Y	N	N	N	N	N
MxA	N	Y	Y	ND	N	ND	Y	Y	Y	Y	Y	N
Treatment‐naïve before MRI	N	Y	Y	N	Y	N	Y	N	Y	Y	Y	Y
Follow‐up time (months)	42	2	16	20	NA	52	18	22	41	36	39	16
Treatment	MPA + AZA	MPA	Prednisone +Tac	Prednisone	Prednisone	Prednisone	Prednisone +Tac	Prednisone	Prednisone +MTX	Prednisone	Prednisone+MTX	Prednisone
Prognosis	Death	Death	Marked improvement	Death	Lost follow‐up	Marked improvement	Marked improvement	Mild improvement	Marked improvement	Death	Marked improvement	Marked improvement

Abbreviations: AZA, azathioprine; Cap, capillary; CK, creatine kinase; DM, dermatomyositis; F, female; ND, not done; M, male; MAC, membrane attack complex; MHC, major histocompatibility complex class; MMT, manual muscle testing; MPA, methylprednisolone aceponate; MRI, magnetic resonance imaging; MTX, methotrexate; MxA, myxovirus resistance protein A; Pt, patient; Sac, sarcolemma; Tac, tacrolimus; and TIF, transcriptional intermediate factor.

Normal serum creatine kinase (CK) levels ranged from 38 to 174 U/L. Anti‐TIF1‐γ antibody antibodies were detected in all patients by immunodot assay (Autoimmune Myositis Profile Antibody IgG Detection Kit MT559, MyBiotech Co., Ltd, Xi'an, China), according to standard methods. Muscle biopsies were taken from the biceps brachii, deltoid, or quadriceps of all 12 involved patients positive for anti‐TIF1‐γ antibody. Serial frozen sections were stained with hematoxylin and eosin (HE), anti‐CD3 mouse monoclonal antibody (clone LN10; Zhongshan Golden Bridge Biotechnology, China), anti‐CD8 rabbit monoclonal antibody (clone SP16; Zhongshan Golden Bridge Biotechnology), anti‐MHC‐I rabbit monoclonal antibody (clone EP1395Y; Abcam, UK), anti‐MHC‐II mouse monoclonal antibody (clone CR3/43; Dako, Denmark), anti‐membrane attack complex (MAC) mouse monoclonal antibody (clone aE11; Dako), and anti‐MxA rabbit polyclonal antibody (Polyclonal, Abcam). Perifascicular necrosis/regeneration, perifascicular atrophy, and the expression of autoimmune markers, including MHC‐I, MHC‐II, MAC, and MxA, with perifascicular enhancement were all regarded as perifascicular changes.

### 2.3 Muscle MRI

Lower limb MRI was conducted using a 3 T MRI system (Verio; Siemens Medical Solutions, Erlangen, Germany). The slices were 5–7 mm thick with a flip angle of 90°, a field of view of 412 × 549 to 864 × 550 mm, a matrix of 252 × 225 to 553 × 553 mm, and an acquisition time of 2:27–2:34 min. The following sequences were used: (1) axial T1‐weighted fast/turbo spin echo series (repetition time (TR) 478 ms; echo time (TE) 10 ms); (2) one of axial fat‐saturated sequences including axial T2‐weighted fast spin echo fat saturation (FS) series (TR 924 ms; TE 80 ms), axial Ideal T2 series (TR 4800 ms; TE 85 ms), and axial spectral attenuated inversion recovery (SPAIR) series (TR 2500 ms; TE 60 ms); and (3) coronal short‐tau inversion recovery (STIR) fast spin echo series (TR 6770 ms; TE 70 ms; inversion time 230 ms sequences).

### 2.4 Image analysis

Edema of the muscular fasciae, subcutaneous tissue, and muscle groups were evaluated using FS/Ideal T2/SPAIR and STIR. The degree of muscle edema was determined using the Stramare scale as follows: scale 0, normal appearance; scale 1, mild, interfascicular increased signal intensity; scale 2, mild, intrafascicular segmented increased signal intensity in less than 50% of the volume of the muscle; scale 3, mild, intrafascicular extensive increased signal intensity in more than 50% of the volume of the muscle; scale 4, moderate, intrafascicular segmented increased signal intensity in less than 50% of the volume of the muscle; and scale 5, moderate, intrafascicular extensive increased signal intensity in more than 50% of the volume of the muscle.^19^ The fatty replacement was evaluated on T1 sequences according to the modified Mercuri's scale as follows: scale 0, normal appearance; scale 1, scattered distribution of increased signal intensity; scale 2, areas of confluent increased signal intensity in less than 30% of the volume of the muscle; scale 3, areas of confluent increased signal intensity in 30%–60% of the volume of the muscle; scale 4, areas with confluent increased signal in more than 60% of the volume of the muscle; and scale 5, muscle entirely replaced by areas of confluent increased signal.^20^, ^21^ Indeed, firstly we evaluated the edema score of each muscle group through the axial position. The scores at the proximal and distal ends were recorded separately. If there was a discrepancy between the scores of the proximal and distal ends, we counted the number of layers affected by the different scores and set the score with the more affected layers as the final edema score of the muscle. Finally, we evaluated the overall edema of this muscle in the sagittal position to ensure the reliability of the edema score.

In this study, we examined the skeletal muscle, muscular fascia, and subcutaneous tissue of the lower limbs, including the muscles of the hip, thigh, and calf. The hip muscles were divided into the following segments: (1) the anterior compartment (tensor fascia latae) and (2) the posterior compartment (gluteus maximus, obturator internus, obturator externus, and quadratus femoris). Similarly, the thigh muscles were divided into (1) the anterior compartment (sartorius, rectus femoris, vastus lateralis, vastus medialis, and vastus intermedius), (2) the medial compartment (gracilis, adductor longus, adductor brevis, and adductor magnus), and (3) the posterior compartment (caput breve and caput longum of the biceps femoris, semimembranosus, and semitendinosus). The calf muscles included (1) the anterior compartment (tibialis anterior, extensor hallucis longus, and extensor digitorum longus), (2) the lateral compartment (peroneus longus and peroneus brevis), and (3) the posterior compartment (caput mediale and caput laterale of the gastrocnemius, soleus, tibialis posterior, flexor hallucis longus, and flexor digitorum longus). An asymmetric MRI pattern was defined as no less than one scale between the two sides of the same muscle group.

Magnetic analysis of MRI image was performed by a musculoskeletal radiologist (JH) and a neurologist (CZ) blinded to the demographic and clinical features. In muscles with different edema and fatty replacement scores, the two readers reviewed the muscles together to agree on the final score.

### 2.5 Statistical analysis

All statistical analyses were performed using the SPSS 26 software. We conducted a normality test using the Shapiro‐Wilk method for all data subjected to statistical analysis. Continuous variables with a normal distribution are expressed as the mean ± standard deviation, whereas those without a normal distribution are presented as the median (quartile). Pearson's correlation analysis was used for continuous variables conforming to a normal distribution, and Spearman's correlation analysis was used for continuous variables not conforming to a normal distribution. Statistical significance was set at *p* < 0.05. Correlation coefficient *r* ≥ 0.8 was regarded as a “strong” correlation. Simple linear regression analysis was conducted to fit a linear regression model for the two continuous variables with correlations.

## 3 RESULTS

### 3.1 Clinico‐pathological features of 12 adult DM patients positive for anti‐TIF1‐γ antibody

**FIGURE 1:**
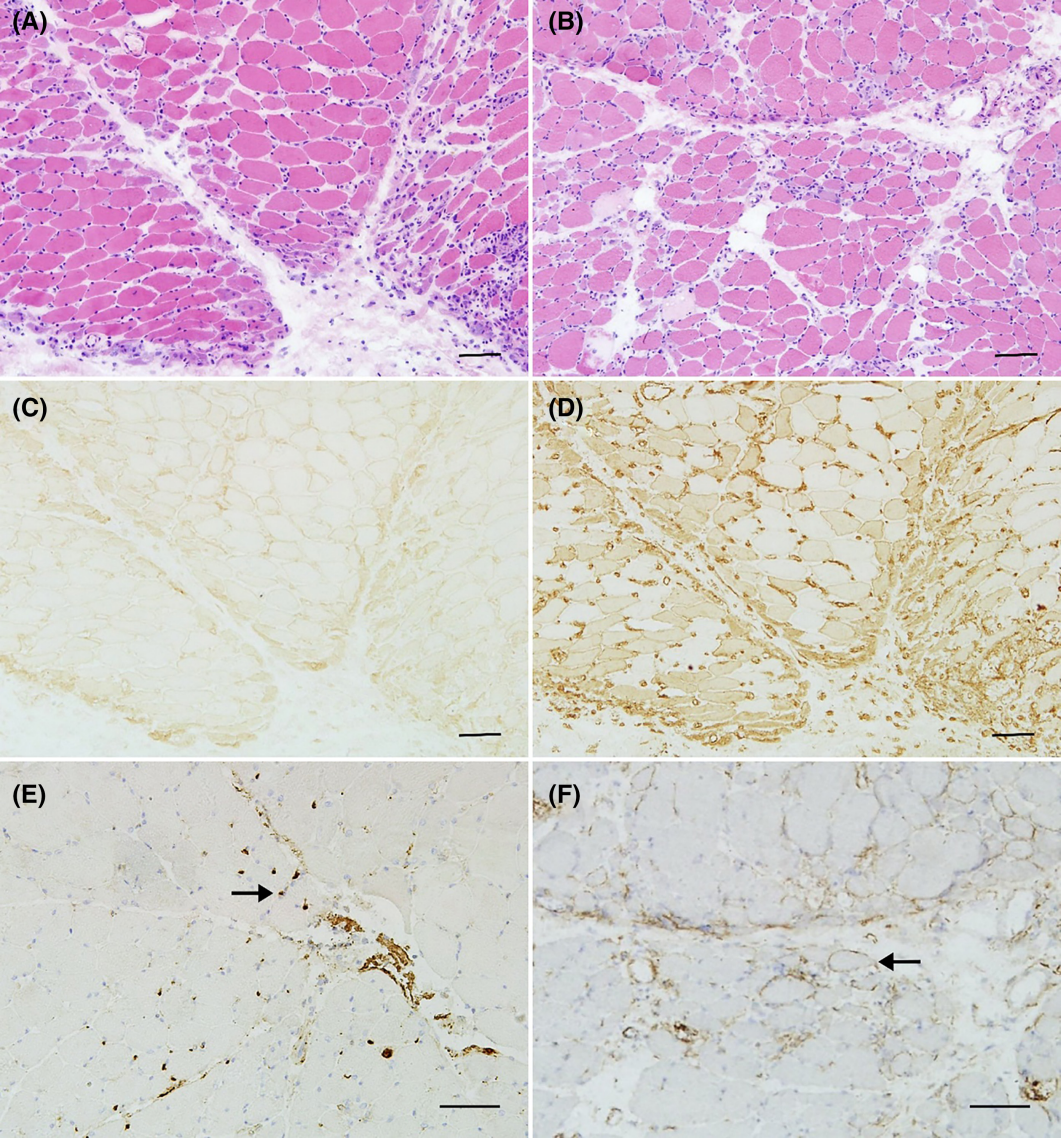
The perifascicular changes of muscle biopsy in patients with anti‐TIF1‐γ antibody. (A) Perifascicular atrophy in Patient 3. (B) Perifascicular necrosis in Patient 6. (C) Perifascicular enhancement of MHC‐I in Patient 3. (D) Perifascicular enhancement of MHC‐II in Patient 3. (E) Perifascicular enhancement of MAC in capillary in Patient 7 (black arrow). (F) Perifascicular enhancement of MAC in sarcolemma in Patient 6 (black arrow). Scale bars = 50 μm. MAC, membrane attack complex; MHC, major histocompatibility complex class; and TIF, transcriptional intermediate factor.

The study enrolled 12 adult DM patients with anti‐TIF1‐γ antibody, of whom 11 were women. The clinical characteristics of the patients are summarized in Table 1. The mean age at disease onset was 57.3 years (SD, 16.7; range 19–76 years), and the median (first and third quartiles) serum CK level at the time of MRI was 537 (162.25–875) U/L. All 12 patients showed weakness in the proximal lower extremities, 1 (8.3%) with asymmetric muscle weakness, 8 (66.7%) with dysphagia, 8 (66.7%) with skin edema, and 3 (25.0%) with malignancies. Muscle biopsy revealed obvious inflammatory cell infiltration in 8 patients (66.7%), perifascicular changes in 10 patients (83.3%) including perifascicular necrosis/regeneration in 1 patient, perifascicular atrophy in 5 patients, and expression of autoimmune markers with perifascicular enhancement in 9 patients (Figure 1). After diagnosis at our center, all patients received glucocorticoid therapy, and five also received immunosuppressants. Four patients died during follow‐up including two with malignancies, and one was lost to follow‐up. Among the remaining 7 patients followed up for more than 16 months (29.1 ± 14.6 months), 6 cases showed marked improvement and 1 showed mild improvement. None of the patients experienced any recurrence.

### 3.2 Edema of the muscular fasciae and subcutaneous tissue in DM patients with anti‐TIF1‐γ antibody

**FIGURE 2:**
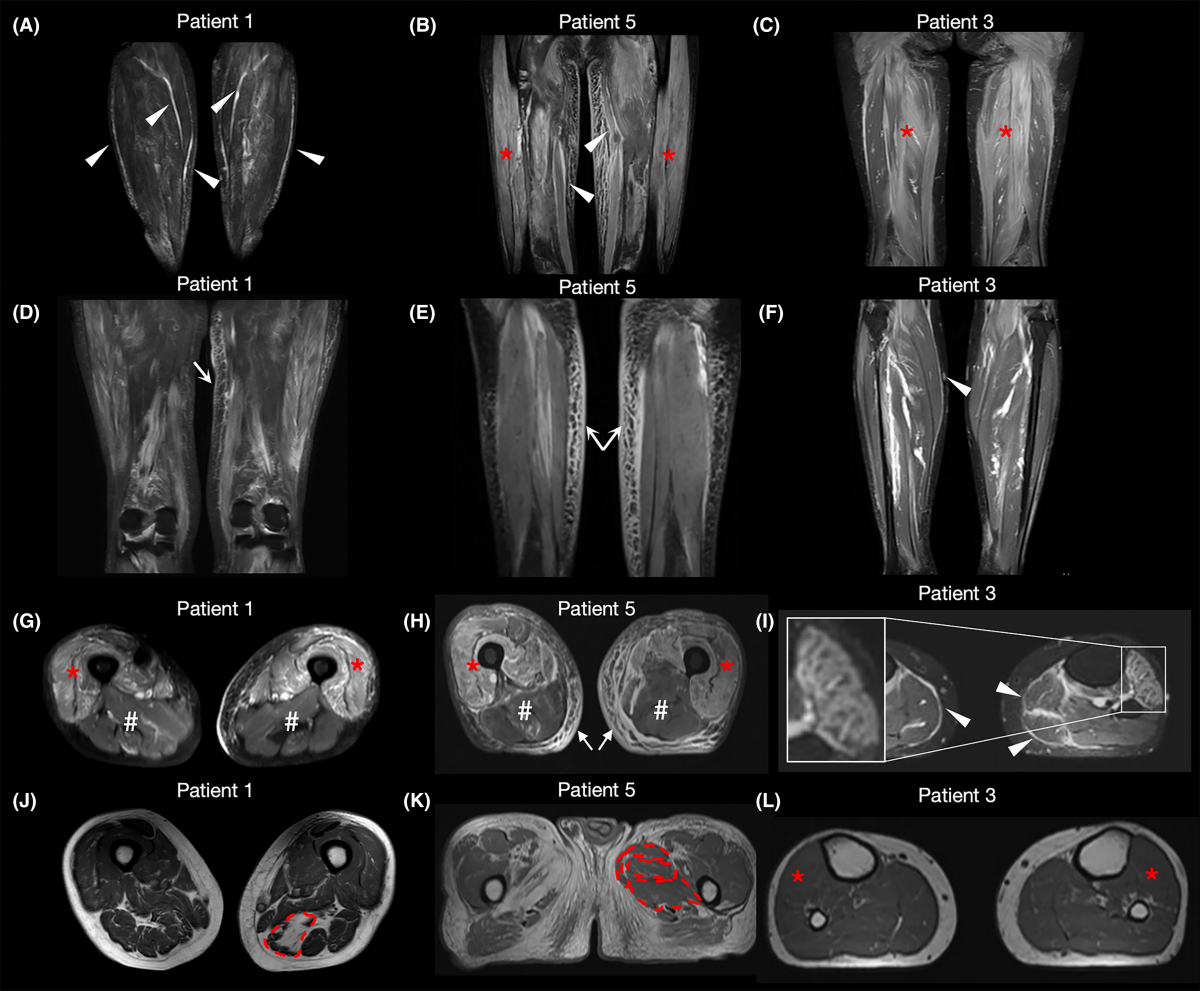
Typical muscle MRI manifestations of patients with anti‐TIF1‐γ antibody. (A, D, G, J) The MRI manifestations of Patient 1. (B, E, H, K) The MRI manifestations of Patient 5. (C, F, I, L) The MRI manifestations of Patient 3. (A) (Coronal STIR), obvious myofascial edema of gastrocnemius, soleus, and peroneus longus is shown (white arrowheads). (B) (Coronal STIR), obvious myofascial edema of the sartorius (white arrowheads) and diffuse edema in the vastus lateralis and vastus intermedius (red stars) are observed. (C) (Coronal STIR), diffuse edema in the gracilis, semimembranosus, and semitendinosus is observed (red stars). (D) (Coronal STIR), diffuse subcutaneous‐tissue edema is observed in the left medial thigh. (E) (Coronal STIR), bilateral symmetrical honeycomb‐like edema of subcutaneous tissue of thigh is shown (white arrows). (F) (Coronal STIR), remarkable myofascial edema of gastrocnemius is observed (white arrowheads). (G) (Axial SPAIR), muscle edema is observed in anterior compartment of the thigh muscle, and that is more severe on the left side (red stars), while the posterior compartment is relatively spared (red #s). (H) (Axial FS) Obvious edema in the anterior compartment of the thigh muscle is observed (red stars), which is more severe on the right side, while the posterior compartment is relatively spared (red #s). Remarkable subcutaneous‐tissue edema is shown (white arrows). (I) (Axial T2‐FS), remarkable myofascial edema of gastrocnemius is observed (white arrowheads), and muscle edema of the anterior compartment showed a honeycomb‐like appearance (solid line box). (J) (Axial T1WI), the fatty infiltration of the left semimembranosus is severe (red dashed lines), while it is moderate on the right side. (K) (Axial T1WI), the fatty infiltration of the right medial compartment of the thigh muscle is severe, with complete replacement of the right gracilis, adductor longus, and adductor brevis muscles by lipids. However, the medial compartment of the left thigh is relatively spared. L (Axial T1WI), fatty infiltration is not evident in the anterior compartment of the calf muscles (red stars). FS, fat saturation; MRI, magnetic resonance imaging; SPAIR, spectral attenuated inversion recovery; STIR, short‐tau inversion recovery; TIF, transcriptional intermediate factor; and T1WI, T1‐weighted imaging.

**FIGURE 3:**
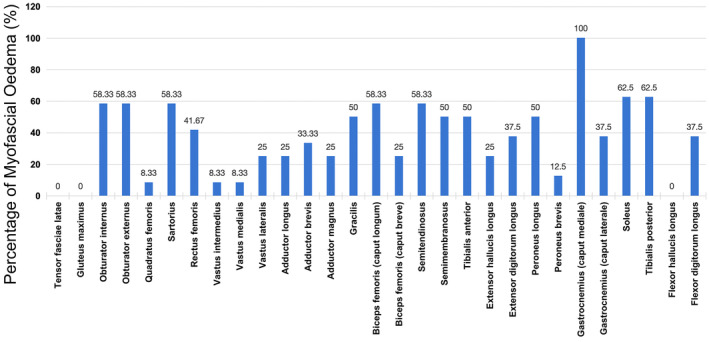
The percentage of myofascial edema in the lower limb muscles of dermatomyositis patients with anti‐TIF1‐γ antibody. TIF, transcriptional intermediate factor.

All 12 patients underwent hip and thigh MRI, and 8 also underwent calf MRI. Before undergoing muscle MRI, five patients were administered methylprednisolone for 2–3 days, while the remaining patients were not administered any glucocorticoids or immunosuppressant therapy. Myofascial edema was found in all patients included in our study (Figure 2A,B,F,I). Among the hip muscles, the obturator internus and obturator externus had the highest proportion (58.3%) of myofascial edema with sparing of tensor fasciae latae and gluteus maximus muscles. Among the thigh muscles, the sartorius, caput longum of the biceps femoris, and semitendinosus showed the highest proportion (58.3%) of myofascial edema. In the calf groups, all eight patients had myofascial edema of the gastrocnemius (caput mediale), and none had myofascial edema of the flexor hallucis longus (Figures 2A,F,I and 3). Moreover, eight patients showed edema of the subcutaneous tissue, with one in the hip, seven in the thigh, and five in the calf (Figure 2D,E,H).

### 3.3 Muscle edema in DM patients with anti‐TIF1‐γ antibody

**FIGURE 4:**
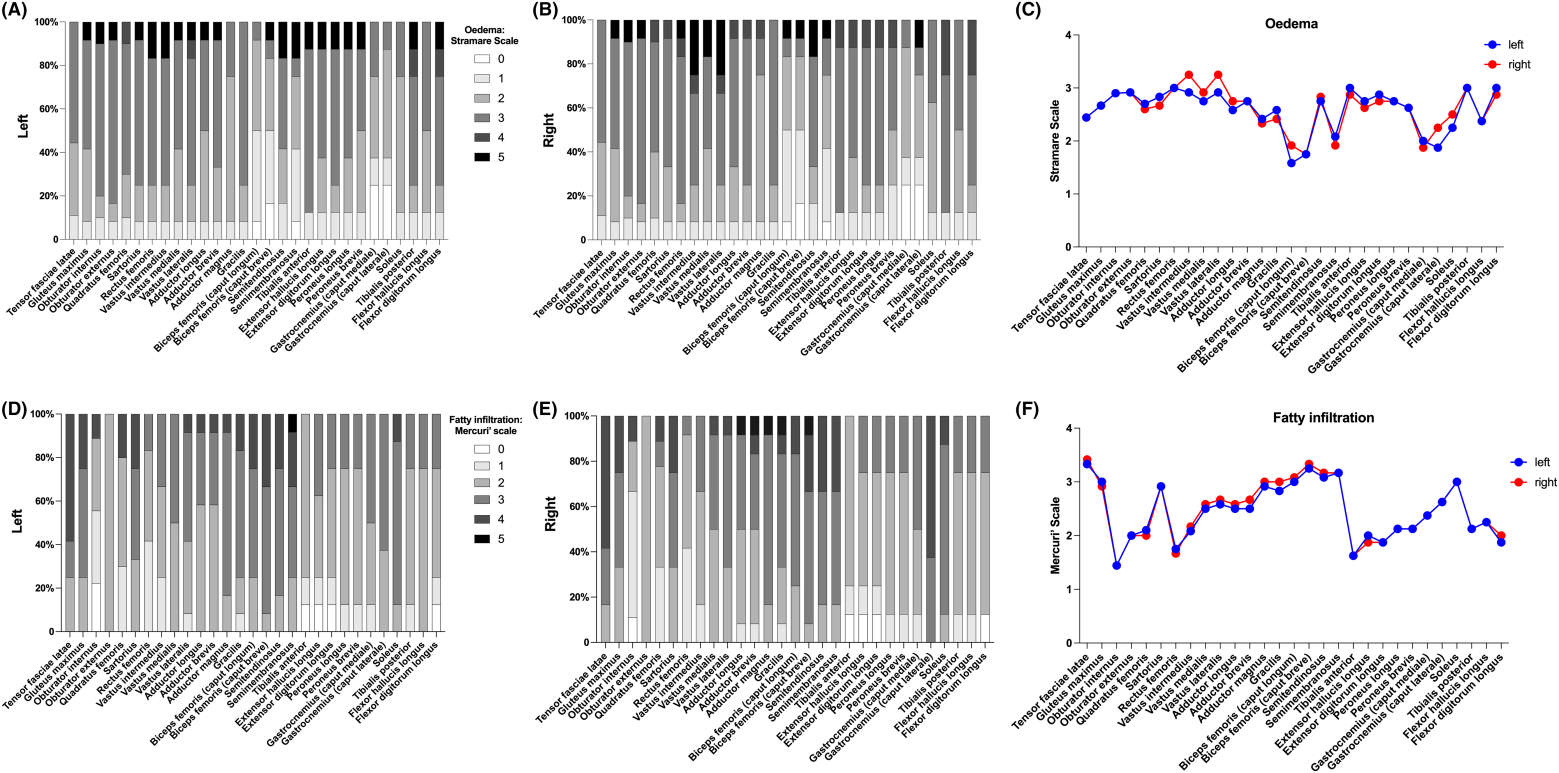
The severity and distribution of muscle edema and fatty replacement in the lower limbs of dermatomyositis patients with anti‐TIF1‐γ antibody. (A) The score for the degree of muscle edema of the left lower limbs (longitudinal axis depicts the percentage of scores across different levels). (B) The score for the degree of muscle edema of the right lower limbs. (C) The mean Stramare scale for muscle edema of lower limb muscles. (D) The score for the degree of fatty infiltration in the muscles of the left lower limbs. (E) The score for the degree of fatty replacement in the muscles of the right lower limbs. (F) The mean Mercuri' scale for fatty replacement of lower limb muscles. TIF, transcriptional intermediate factor.

All 12 enrolled patients had muscle edema in the lower limbs, and most muscles showed an edema scale of 2–3 (Figures 2B,C,G–I and 4A–C). Among the hip muscles, the obturator internus and the obturator externus had the most severe edema. Among the thigh groups, the anterior compartment had the most severe edema, followed by the medial and posterior compartments. Specifically, the vastus intermedialis and vastus lateralis showed the most severe edema, whereas the caput longum and breve of the biceps femoris and semimembranosus were relatively spared. Among all the calf muscles, the caput mediale and caput laterale of the gastrocnemius were relatively spared, whereas the other groups were similarly affected (Figure 4A–C). Asymmetric muscle edema was found in nine patients, including one with asymmetric muscle weakness (Figure 2G,H). No cases of muscle attachment point edema were observed.

### 3.4 Fatty replacement in DM patients with anti‐TIF1‐γ antibody

All 12 enrolled patients had fatty replacement, with all displaying varying degrees of fatty replacement among the different muscle groups (Figures 2J–L and 4D–F). Among the hip muscles, the tensor fasciae latae and gluteus maximus muscles showed the most severe fatty replacement. Among the thigh muscles, the caput breve of the biceps femoris and semitendinosus muscles showed the most severe fatty replacement, whereas that of the rectus femoris was relatively spared. The soleus exhibited the highest fatty replacement, followed by the caput mediale and caput laterale of the gastrocnemius in calf muscles (Figure 4D–F). Asymmetric fatty replacement was observed in 10 patients, including 1 with asymmetric muscle weakness (Figure 2J,K). Moreover, correlation analysis showed that the degree of muscle edema and fatty replacement in the lower limbs were not parallel (*p >* 0.05) (Figure 4C,F).

### 3.5 Correlations between MRI changes and clinical data

A positive correlation was found between the edema score of the posterior compartment of the thigh muscles and disease duration (*p* = 0.048, *r* = 0.581). Moreover, edema changes, including the global edema score of the thigh muscles, the edema score of the medial compartment of thigh muscles, the global edema score of calf muscles, and edema score of the posterior compartment of calf muscles, were positively correlated with CK levels (*p* = 0.015, *r* = 0.678; *p* = 0.001, *r* = 0.832; *p* = 0.045, *r* = 0.719; and *p* = 0.017, *r* = 0.80, respectively) (Table 2). No significant relationship was found between MRI changes including muscle edema and fatty replacement and treatment outcomes (*p* > 0.05) (Table S1). There was no significant correlation between fatty MRI changes including the global score of fatty replacement, scores of each compartment, and clinical manifestation including age at onset, disease duration, and CK levels (*p* > 0.05) (Table 2).

**TABLE 2:** The correlation analysis between muscle MRI changes and clinical data.

	Duration of disease	Age of onset	CK
Edema scores of hip muscles			
Global	0.628	0.692	0.166
Anterior compartment	0.789	0.637	0.733
Posterior compartment	0.480	0.745	0.098
Edema scores of thigh muscles			
Global	0.985	0.781	0.015* (*r* = 0.678)
Anterior compartment	0.271	0.479	0.094
Medial compartment	0.161	0.670	0.001** (*r* = 0.832)
Posterior compartment	0.048* (*r* = 0.581)	0.693	0.702
Edema scores of calf muscles			
Global	0.644	0.463	0.045* (*r* = 0.719)
Anterior compartment	0.976	1	0.199
Lateral compartment	0.810	0.571	0.156
Posterior compartment	0.597	0.387	0.017* (*r* = 0.802)
Fatty replacement scores of hip muscles			
Global	0.281	0.738	0.664
Anterior compartment	0.864	0.212	0.799
Posterior compartment	0.234	0.473	0.484
Fatty replacement scores of thigh muscles			
Global	0.095	0.108	0.484
Anterior compartment	0.108	0.274	0.688
Medial compartment	0.270	0.082	0.237
Posterior compartment	0.129	0.192	0.178
Fatty replacement scores of calf muscles			
Global	0.444	0.156	0.866
Anterior compartment	0.338	0.349	0.684
Lateral compartment	0.472	0.485	0.696
Posterior compartment	0.546	0.298	0.866

Abbreviation: CK, creatine kinase.

* *p* < 0.05.

** *p* < 0.01.

Besides, there was no statistically significant difference in the fatty and edema scores of muscle groups between patients with tumors and those without tumors (*p* > 0.05, Table S2), and no difference was found between these two groups in field of the frequencies of myofascial edema and subcutaneous‐tissue edema.

## 4 DISCUSSION

This is the first study to evaluate the pattern of edema and fatty replacement changes of anti‐TIF1‐γ antibody‐positive patients in a Chinese case series. Overall, we find that myofascial edema of the gastrocnemius was a prominent MRI characteristic of anti‐TIF1‐γ‐positive DM. Our analysis also showed that these anti‐TIF1‐γ‐positive patients showed a distinct pattern of muscle edema and fatty replacement changes of lower limbs with common asymmetric pattern in MRI. The vastus intermedialis and vastus lateralis showed the most severe muscle edema, while the tensor fasciae latae, biceps femoris, semitendinosus, and soleus muscles showed the most severe fatty replacement.

Anti‐TIF1‐γ antibody is a multifunctional protein related to immunoregulation, cell cycle, and transcription of a tumor suppressor gene.^22^ This antibody has a relatively high frequency (approximately 25%) in adults with DM and is closely associated with malignancy.^3^, ^5^, ^23^ In our study, three of the enrolled patients (25%) showed malignancy, and two (67%) died during follow‐up. In view of the high prevalence of malignancy in patients with anti‐TIF1‐γ‐positive DM and the poor prognosis in patients with malignancy, the early diagnosis of anti‐TIF1‐γ‐positive DM is very important. Unfortunately, among patients with anti‐TIF1‐γ antibody, MRI features were not correlated to malignancies. Since myofascial edema of the gastrocnemius was found in all our tested patients and a high frequency of gastrocnemius myofascial edema was not reported in any other group of IIMs, we assumed that this unique MRI finding could be helpful in the early diagnosis of anti‐TIF1‐γ antibody‐positive DM to monitoring malignancy occurrence.

All of the patients included in our study had myofascial edema, consistent with previous findings that high signal intensity of the fascia is a characteristic MRI feature of DM.^24^, ^25^ We further detected the unique pattern of myofascial edema in patients with anti‐TIF1‐γ antibody as prior studies did not focus on the myofascial edema patterns of each kind of MSA. Specifically, myofascial edema of the calf muscles was found in all of our patients, while myofascial edema of the hip and thigh muscles was also found in most patients. Since most of our patients with anti‐TIF1‐γ antibody showed perifascicular changes as reported, we inferred that the high frequency of myofascial edema involvement in anti‐TIF1‐γ antibody‐positive DM may correspond to the obvious perifascicular damage in muscle biopsy.^5^, ^8^ The remarkable myofascial edema changes potentially implied the important role of perifascicular changes in the pathogenesis of anti‐TIF1‐γ antibody‐positive DM. This view was supported by another report in China, which found that patients with anti‐synthetase syndrome (ASS) characterized by perimysial connective tissue in muscle pathology also exhibited frequent myofascial edema.^9^ Indeed, the pattern of myofascial edema was significantly different between the two subgroups. Specifically, patients with ASS usually showed myofascial edema of the tensor fasciae latae, while our patients with anti‐TIF1‐γ antibody showed myofascial edema of gastrocnemius without tensor fasciae latae involvement.^9^ This difference may be useful for distinguishing between two similar subgroups.

Unlike IIMs with antibodies to signal recognition particle (SRP) which show more pronounced edema than fatty replacement, no significant difference was shown between the degree of fatty replacement and muscle edema in patients with anti‐TIF1‐γ antibody.^15^ Moreover, the patterns of fatty replacement and muscle edema differed between the two groups. Specifically, the vastus lateralis, rectus femoris, biceps femoris, and adductor magnus were the muscles of most severe edema affected in anti‐SRP myopathy, while the vastus intermedialis and vastus lateralis were the most affected in DM patients with anti‐TIF1‐γ antibody.^15^ The most severe fatty replacement muscles were the hamstrings and adductor magnus in anti‐SRP myopathy, while the biceps femoris and semitendinosus were the most affected in anti‐TIF1‐γ antibody‐positive DM. The different MRI findings between these two subgroups support our finding that each type of MSA has unique MRI characteristics.

Strikingly, asymmetric MRI pattern was common in our anti‐TIF1‐γ antibody‐positive patients. Unlike inclusion body myositis with marked asymmetric involvement, most patients show subclinical asymmetric muscle weakness.^26^ Since patients with ASS and patients with immune‐mediated necrotizing myopathy also showed bilateral asymmetry in previous reports, we assumed that the involvement of the muscle groups was not strictly symmetrical in IIM.^9^, ^10^

Since CK is the most sensitive indicator of myositis and its elevation is often parallel to the degree of muscle injury to some extent, it is generally seen as an indicator of myopathy activity.^27^ We further found that MRI changes in muscle edema were related to CK levels. Muscle edema features, including the global edema score of the thigh muscles, global edema score of the calf muscles, edema score of the medial compartment of thigh muscles, and the posterior compartment of the calf muscles, were all positively correlated with CK levels in our study. The relationship between the degree of edema and CK levels was not observed in anti‐SRP myopathy, whereas the total edema score was correlated with the CK level in ASS subtype.^15^, ^28^ This inconsistent association between muscle edema and CK levels among studies may also support the view that each MSA type has distinct MRI features. We found no significant relationship between fatty replacement and disease duration in anti‐TIF1‐γ‐positive DM in our analysis, which differed from sporadic inclusion body myositis (sIBM) and anti‐SRP myopathy. Studies have shown that the degree of fatty replacement is positively correlated with disease duration in both sIBM^29^ and anti‐SRP myopathy.^15^ We hypothesized that this difference was partly related to the fact that anti‐TIF1‐γ‐positive DM tended to show an acute or subacute course, whereas sIBM tended to have a chronic course. Fatty replacement is thought to be a compensatory response to muscle lesions and may lead to chronic stage.^15^ Besides, no relationship was observed between fatty replacement and treatment outcomes.

The primary limitation of the present study is the lack of comparison among different types of MSAs to detect the distinguish MRI feature of patients with anti‐TIF1‐γ antibody. As all tested anti‐TIF1‐γ antibody‐positive patients showed myofascial edema of gastrocnemius, we deem that this finding could be regarded as a prominent MRI characteristic of anti‐TIF1‐γ‐positive DM. Longitudinal studies on individual patients should be performed to explore the role of MRI changes in monitoring disease progression and treatment responses.

In conclusion, myofascial edema of gastrocnemius was the most characteristic MRI change of DM with anti‐TIF1‐γ antibody. These patients often showed that the anterior compartment had the most severe muscle edema and the posterior compartment had the most severe fatty infiltration in the thigh. Moreover, asymmetric MRI patterns were commonly observed. Early detection of muscle edema, as well as CK levels, may be helpful for monitoring disease activity. Given that this study is a retrospective review of a relatively small sample of anti‐TIF1‐γ‐positive DM, further comparison studies among different kinds of MSA based on larger cohorts of patients are required to confirm our conclusions.

## FUNDING INFORMATION

This study was supported by National Natural Science Foundation of China (Grant nos. 82201556; 82071412; and 82171395) and Natural Science Foundation of Shandong Provincial (Grant nos. ZR2021QH120).

## CONFLICT OF INTEREST STATEMENT

The authors declare that there is no conflict of interest.

## Supporting information


Figure S1.

Table S1.

Table S2.

## Data Availability

The datasets used and analyzed during the current study are available from the corresponding author on reasonable request.
