# Supplementary figures and images for: Myofascial edema of gastrocnemius: A prominent MRI characteristic in dermatomyositis patients with anti‐transcriptional intermediate factor 1‐γ antibody
Source: CNS Neurosci Ther. 2024 Feb 22;30(2):e14647. doi: 10.1111/cns.14647 (PMC10883095; doi:10.1111/cns.14647)

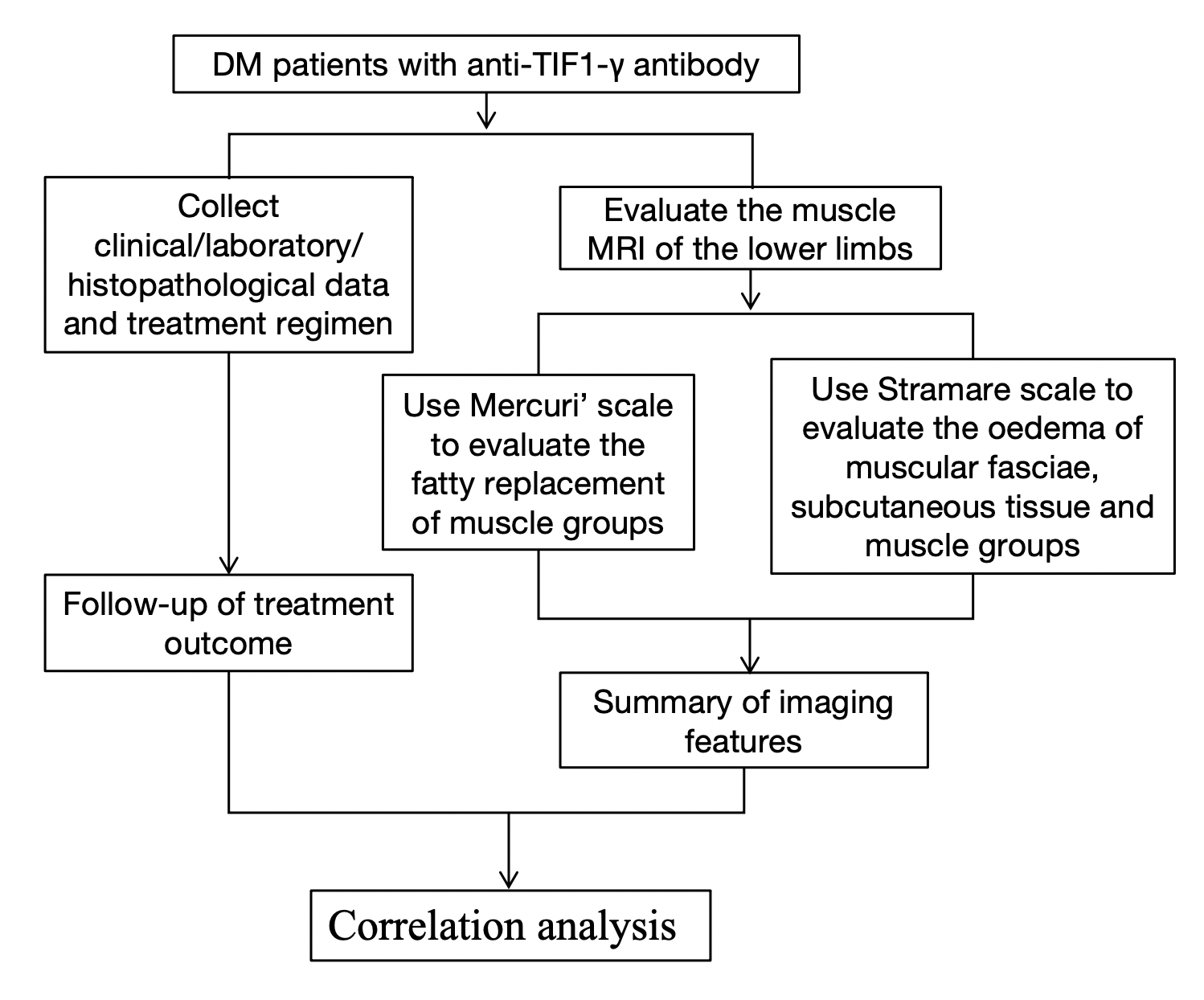

Supplement: Supplementary file 1 — Figure S1. [file CNS-30-e14647-s002.tif]
