# Supplementary material for: Myofascial edema of gastrocnemius: A prominent MRI characteristic in dermatomyositis patients with anti‐transcriptional intermediate factor 1‐γ antibody
Source: CNS Neurosci Ther. 2024 Feb 22;30(2):e14647. doi: 10.1111/cns.14647 (PMC10883095; doi:10.1111/cns.14647)
Supplement: Supplementary file 2 — Table S1. [file CNS-30-e14647-s001.docx]

Supplementary Table 1 The comparison of the fatty and oedema scores of muscle groups between anti-TIF1-γ antibody-positive DM patients with malignancies and without malignancies.

|  | Anti-TIF1-γ -positive DM with malignancies | Anti-TIF1-γ-positive DM without malignancies | *p* |
| --- | --- | --- | --- |
| Global score of muscle oedema |  |  |  |
| hip muscles (mean ± SD) | 29.33±1.15 | 22.33±9.64 | 0.252 |
| thigh muscles (mean ± SD) | 73.67±7.64 | 63.44±18.21 | 0.379 |
| calf muscles (mean ± SD) | 66.33±19.60 | 48±15.98 | 0.196 |
| Global score of fatty replacement |  |  |  |
| hip muscles (mean ± SD) | 16.33±4.04 | 22.89±7.06 | 0.165 |
| thigh muscles (mean ± SD) | 66±12.49 | 72.44±13.78 | 0.491 |
| calf muscles (mean ± SD) | 40±12.17 | 52.8±12.03 | 0.197 |
| Oedema of the muscular fascia (n, %) | 3, 100% | 9, 100% | 1.00 |
| Oedema of subcutaneous tissue (n, %) | 2, 66.67% | 6, 66.67% | 1.00 |

Abbreviations: DM, dermatomyositis; SD, standard deviation; TIF1-γ, transcriptional intermediate factor 1-γ.
