# Supplementary material for: Myofascial edema of gastrocnemius: A prominent MRI characteristic in dermatomyositis patients with anti‐transcriptional intermediate factor 1‐γ antibody
Source: CNS Neurosci Ther. 2024 Feb 22;30(2):e14647. doi: 10.1111/cns.14647 (PMC10883095; doi:10.1111/cns.14647)
Supplement: Supplementary file 3 — Table S2. [file CNS-30-e14647-s003.docx]

Supplementary Table 2 The correlation analysis between muscle MRI changes and treatment outcomes.

|  | Treatment outcomes (*p*) |
| --- | --- |
| Global score of muscle oedema |  |
| hip muscles | 0.363 |
| thigh muscles | 0.988 |
| calf muscles | 1.000 |
| Global score of fatty replacement |  |
| hip muscles | 0.231 |
| thigh muscles | 0.172 |
| calf muscles | 0.170 |

Abbreviations: MRI, magnetic resonance imaging.
